# Magnitude and associated factors of disrespect and abusive care among laboring mothers at public health facilities in Borena District, South Wollo, Ethiopia

**DOI:** 10.1371/journal.pone.0256951

**Published:** 2021-11-18

**Authors:** Mulusew Maldie, Gudina Egata, Muluken Genetu Chanie, Amare Muche, Reta Dewau, Nigusu Worku, Mamo Dereje Alemu, Gojjam Eshetie Ewunetie, Tesfaye Birhane, Elsabeth Addisu, Wolde Melese Ayele, Metadel Adane

**Affiliations:** 1 Borena Woreda Health Office, South Wollo Ethiopia, Borena, Ethiopia; 2 Schools of Public Health, Haramaya University, Harar, Ethiopia; 3 Department of Health Systems and Policy, School of Public Health, College of Medicine and Health Sciences, Wollo University, Dessie, Ethiopia; 4 Department of Epidemiology and Biostatistics, School of Public Health, College of Medicine and Health Sciences, Wollo University, Dessie, Ethiopia; 5 Department of Health Systems and Policy, Institute of Public Health, College of Medicine and Health Sciences, University of Gondar, Gondar, Ethiopia; 6 Health Systems Strengthening Directorate, Federal Ministry of Health, Addis Ababa, Ethiopia; 7 Department of Human Resource for Health, Denbya Primary Hospital, Gondar, Ethiopia; 8 Department of Reproductive Health, School of Public Health, College of Medicine and Health Sciences, Wollo University, Dessie, Ethiopia; 9 Department of Environmental Health Sciences, College of Medicine and Health Sciences, Wollo University, Dessie, Ethiopia; Medical Research Council, SOUTH AFRICA

## Abstract

**Background:**

Recent studies have indicated that disrespectful/abusive/coercive service by skilled care providers in health facilities that results in actual or perceived poor quality of care is directly and indirectly associated with adverse maternal and newborn outcomes. According to the 2016 Ethiopian Demography and Health Survey, only 26% of births were attended by qualified clinicians, with a maternal mortality rate of 412 per 100,000 live-births. Using seven categories developed by Bowser and Hill (2010), this study looked at disrespect and abuse experienced by women in labor and delivery rooms in health facilities of Borena Ddistrict, South Wollo, Ethiopia.

**Methods:**

A facility-based cross-sectional study was conducted among 374 immediate postpartum women in Borena District from January 12 to March 12, 2020. Systematic sampling was used to access respondents to participate in a structured, pre-tested face-to-face exit interview. Data were entered into EpiData version 4.6 and exported to SPSS version 25 for analysis. Finally, bivariable and multivariable logistic regression analysis were performed to declare statistically significant factors related to maternal disrespect and abusive care in Borena District at a p-value of < 0.05 and at 95% CI.

**Result:**

Almost four out of five (79.4%) women experienced at least one type of disrespect and abuse during facility-based childbirth. The most frequently reported type of disrespect and abuse was non-consented care 63.7%. Wealth index [AOR = 3.27; 95% CI: (1.47, 7.25)], type of health facility [AOR = 1.96; 95% CI: (1.01, 3.78)], presence of companion(s) [AOR = 0.05; 95% CI: (0.02, 0.12)], and presence of complications [AOR = 2.65; 95% CI: (1.17, 5.99)] were factors found to be significantly related to women experiencing disrespect and abuse.

**Conclusion:**

The results showed that wealth index, type of health facility, presence of companion(s), and birth complications were found to be significant factors. Therefore, health personnel need to develop interventions that integrate provider’s behavior on companionship and prevention of complications across facilities to reduce the impact of disrespectful and abusive care for laboring women.

## Background

Disrespect and abuse (D&A) is described as mistreatment, obstetric violence, or dehumanized care, and is a violation of a woman’s rights during maternity care [1]. According to Brower and Hills, disrespect and abuse is divided into seven major categories: physical abuse, non-confidential care, non-dignified care, non-consented care, discrimination, detention, abandonment/neglect of care [2, 3].

Because motherhood is particular to women, issues of gender equity sit at the core of maternity care. It is essential that the idea of safe motherhood be extended beyond the prevention of morbidity or mortality to include respect for women’s basic human rights, including respect for women’s autonomy, dignity, feelings, choices, and preferences, including decisions about who is present at a birth [4, 5].

A key component of the strategy to lessen maternal morbidity and mortality has been to increase numbers of skilled birth attendants and facility-based childbirths. While global skilled birth attendance rates have risen by 12% in developing regions over the past two decades, almost one-third of women in these regions still deliver without a skilled birth attendant [6]. For that reason, maternal health efforts worldwide are shifting from an emphasis on boosting service utilization to improving quality of care. This alteration has been supplemented by a emergent body of work on respectful maternity care during facility-based childbirth [7].

Respectful maternity care (RMC) is a universal human basic right that is due to every childbearing woman in every health system around the world and to be provided to all women in a way that preserves their dignity, privacy, and confidentiality, safeguards freedom from harm and mistreatment, and empowers informed choice and uninterrupted care during maternity care [8].

Globally, RMC is gaining much-deserved recognition and attention from experts working in the field of reproductive health. Disrespect and abuse in maternal and child health care delivery prevents women from seeking proper health services [1]. The laboring process is an experience with deeply personal, cultural, and psychological significance, and because motherhood is specific to women, gender equity and gender violence are also at the core of maternity care [9].

Even though significant improvement in maternal health was observed between 1990 and 2015, maternal mortality continues to be a public health problem globally. In 2015, an estimated 303,000 women lost their lives due to easily avoidable pregnancy and childbirth-related problems worldwide, 99% of which occurred in low- and middle-income countries [10].

In sub-Saharan Africa (SSA), over 162,000 women still die each year during pregnancy and childbirth. Most of those deaths are within 24 hours after delivery [11]. The Ethiopian maternal mortality rate (MMR) is one of the highest in the world. According to EDHS, about 412 mothers die for each 100,000 live births in Ethiopia whereby the majority of deaths occur within 48 hours after delivery [12].

Globally, maternity care often fails to go beyond the prevention of morbidity or mortality to encompass respect for women’s basic human rights. For example, 56%, 50%, and 46% of respondents in one study reported a lack of privacy, lack of informed consent, and verbal abuse, respectively [13]. In Africa, 62% of health providers do not explain procedures to women before delivery [14]. In SSA countries, the overall prevalence of D & A reported across the five studies ranged from 15% to 98% [15]. Moreover, studies done in Ethiopia revealed rates of D&A that ranged from 40% to 92.5% and that approximately 4% to 39% women were disrespected and abused specifically by non-consented care (lack of informed consent) before delivery procedures and abandonment/neglect of care (women is left without care/attention) respectively [14, 16–19].

Disrespect and abuse of women during facility-based maternity care has multiplicative effects. First, it can lead to a human rights violation; second, it can prevent women from utilizing maternal health services in health facilities; third, it may also erode satisfaction and trust in the health system and lead to poor pregnancy outcomes [6, 20–22] On the other hand, respectful maternity care can contribute to the timely provision of care, improved patient–provider communication, and increased adherence to treatments and maternal health service utilization, all of which can improve maternal and neonatal effects [22–25].

The World Health Organization (WHO) quality-of-care dimension recommends respectful maternal care during facility-based maternity care as a key strategy for improvement of quality of maternity services, to reduce disrespect and abuse as well as maternal mortality and morbidity [26]. But evidence shows that there is increasing disrespect and abuse and mistreatment of women during labor, delivery, and post-delivery and this is the main barrier to seeking skilled maternity care throughout the world [15, 22, 27].

Although there has been an improvement in maternity health services in Ethiopia—antenatal care (ANC) coverage increased from 62% to 74% however, only 34% of new mothers attended postnatal skilled care, a rate that is still low [12, 28]. It shows that maternal health service access only not guaranteed for ensured maternal satisfaction and quality care during childbirth and postnatal care without quality health care and maintain women’s right to health [29, 30].

In addition to geographic, financial, and cultural barriers to care, disrespect and abuse are serious obstacles to utilization of maternal health services. They are associated with women’s feelings of being overlooked, being informed of difficult news without proper preparation, being subjected to repetitive examinations without a reason being properly communicated, companions being disallowed, and being left unattended during facility-based maternity care [31]. Moreover, disrespect and abuse during childbirth are attributed to service delivery level factors such as lack of infrastructure and lack of responsibility mechanisms in the facility, and individual-level factors including the mothers’ socio-demographic characteristics, lack of autonomy, obstetric history and the provider’s perspectives [32–38].

Access to health care facilities has radically improved over the past in Ethiopia. However, the utilization of maternal health care services has remained significantly low [12, 28, 39]. Therefore, the provision of compassionate and respectful maternity care is one of the key factors to promote use of facility-based maternity care [26]. Currently, the Ethiopian federal minister of health (FMOH) aspires to provide compassionate and respectful maternity care (CRC) by reducing the prevalence of disrespect and abuse during delivery and postpartum care in health facilities [40, 41]. Nevertheless, the magnitude of disrespect and abuse and associated factors during facility-based childbirth in Ethiopia is not well known; this study was focused on adding its findings to this knowledge.

## Methods and materials

### Study design and setting

A facility-based cross-sectional study was conducted in Borena public health facilities from January 12 to March 12, 2020. Borena is one of the 23 districts (woredas) in the South Wollo zone. It is surrounded by other districts, by Leganbo to the east, Gojjam to the west, Wagdi to the south, and Saint to the north. The total land area of Borena is 5560km^2^ of which 65% is highland area. The total population was 202,832 in 2012 E.C. (Ethiopian Calendar) as projected from the 2007 census. In Borena is the town of Mekane-Selam, which is located 180 km from Dessie (the city of South Wollo); 281 km from Bahir-Dar (the center of the Amhara region) and 581 km from Addis Ababa (the capital city of Ethiopia). There are 39 *kebeles* (4 urban and 35 rural) and one primary hospital, 7 health centers (one urban and 6 rural), and 36 health posts. A total of 5,283 mothers gave birth in Borena health facilities in the 2011 budget year (Borena Woreda Health Office Annual Report, 2011).

### Population

All women living in Borena were the source population of the study while all women who gave birth in selected public health facilities of Borena during the data collection period were used as the study population. Those women who were critically ill and referred to others health facilities were excluded from the study.

### Sample size determination and sampling procedures

The sample size required for the first specific objective was calculated based on a single population proportions formula with the following assumptions: Prevalence of disrespect and abuse during labor and delivery 67.1% [17], 95% confidence level, 10% non- response, 5% margin of error.


$$n=\frac{(Z\alpha /2)2.p.q}{{\left(d\right)}^{2}}\;=\;n=\frac{(1.96)2*0.671*32.9}{{\left(0.05\right)}^{2}}$$

Thus, the final sample size found was 374 after adding 10% non-response rate.

The sample size for the second specific objective was determined using Epi-info version 7.1 software by considering three variables from previous studies but the final sample sizes determined by this software were less the above sample size determined (374). Therefore, the final sample size used for this study was 374.

In Borena there were 8 public health facilities, of which 5 (Mekane-Selam Hospital (187) n1 = 185; Mekane-Selam Health Center (30) n2 = 29; Tewa Health Center (45) n3 = 44; Galemot Health Center (60) n4 = 58; and Dilfrie Health Center (60) n5 = 58) were selected for the study by simple random sampling. From these five public health facilities, the delivery numbers from February and March, 2011 E.C. were taken from the facilities’ registration books and then the calculated sample size was proportionally allocated based on number of deliveries in those two months in each facility. Finally, using systematic random sampling, every 3^rd^ woman who was eligible and available during the data collection period was included in an exit interview at each respective health facility.

### Study variables

The dependent variable of this study was disrespect and abuse (yes/no) while the independent variables were socio-demographic related factors including age, residence, marital status, religion, educational background, economic status; obstetric history including ANC follow-up, parity, complications during labor and delivery, time of delivery, mode of delivery; health care providers- and facility type-related factors including sex of health care provider, type of health facilities, type of health professionals; and individual-related factors including HIV status, history of previous institutional delivery, intention to deliver at health facilities, presence of companion, and decision making power on health issues.

### Operational definitions

Disrespect and abuse (D & A) were measured by seven performance standards (types of disrespect and abuse) including physical abuse, non-consented care, non-confidential care, non-dignified care, discriminated care, neglect of care, and detention in the health facility. Accordingly, those women who replied ‘yes’ to at least one form of disrespect and abuse were labeled as being subjected to disrespect and abuse during labor and delivery services. A total of 22 verification criteria of D & A were used to measure the seven performance standards in the composite scale [42].

#### Physical abuse

Women who were not protected from physical harm or ill-treatment [2]. Measured by 5 verification criteria including 1. A health provider physically hit or slapped, pinched or pushed the mother and/or 2. A health provider verbally insulting the mother and/or 3. A health provider restricted the mother from drinking any fluid throughout the labor course unless medically necessitated and/or 4. The birth attendant pushed a mother’s tummy down to deliver the baby (used fundal pressure) and/or 5. The care providers did not allow the mother to assume the position of her choice during the birth. A woman who answered ‘yes’ to at least one criterion was considered to have been physically abused at the time of childbirth.

#### Non-consented care

Women’s right to information, informed consent, and choices/preferences were not protected. Measured by 5 standard verification criteria including 1. The care provider did not introduce themselves and greet mother and her support person and/or 2. The provider did not encourage the mother and her companion to ask questions and/or 3. The provider did not respond to the mother’s question with politeness and/or 4. The provider did not explain what was being done and what to expect throughout labor and during examinations; and/or 5. a health care provider did not obtain consent or permission before any procedure. If a woman answered ‘yes’ to at least one of the criteria, she was considered as having been abused by non-consented care at the time of childbirth.

#### Non-confidential care

A woman’s confidentiality and privacy was not protected, measured by 3 criteria including 1. The provider did not use drapes or covering to protect the mother’s privacy during any procedure and/or 2. Health providers discussed a mother’s private health information in a manner that others might hear and/or 3. Other persons apart from the care providers were allowed in the room while the mother was giving birth who were able to observe a mother while she was naked on the bed. If A woman answered ’yes’ to at least one of the above criteria, she was considered to have been abused by non-confidential care at the time of childbirth.

#### Non-dignified care

A woman w not treated with dignity and respect, measured by 3 criteria. 1. A health provider shouted at or scolded the mother and/or 2. The care provider blamed the mother for getting pregnant or for shouting/crying due to the pain of delivery and/or 3. The care providers did not allow the mother’s companion to enter the delivery room. If a woman answered ’yes’ to at least one of the criteria, she was considered as being abused by non-dignified care at the time of childbirth.

#### Discrimination

A woman received not equitable care, not free from discrimination. Measured by 2 criteria. If 1. A health care provider discriminated by ethnicity, religion, age, and being rural and/or 2. A health care provider discriminated by HIV-positive status. If a woman answered ’yes’ to at least one of the criteria, she was considered to have been abused in discriminatory care at the time of childbirth.

#### Abandonment/neglect of care

A woman did not get timely care or was left alone without care, measured by 3 criteria. 1. A health provider ignored a mother’s call for help and/or 2. A mother was alone when she gave birth in the health institution because the care providers were not around her and/or 3. A mother encountered a life-threatening condition for which she had shouted for help but could not get anyone to reach her in time. A woman who answered ’yes’ to at least one of the criteria was considered to have been abused by abandonment/neglect of care at the time of childbirth.

#### Detention in a health facility

A woman was detained or confined against her will, measured by 1 criterion 1. Health care providers detained a mother in the health facility because of payment issues when she had posed damage to the property of the health institution. A woman who answered ’yes’ to this criterion was considered to have been abused by detention at the time of childbirth.

#### Wealth index

The wealth index is a composite measure of a household’s cumulative living standard. The wealth index is calculated using easy-to-collect data on a household’s ownership of selected assets, such as televisions, radio, materials used for housing construction; and types of water access and sanitation facilities; pets and others.

### Data collection tools and procedures

The outcome variable was measured using seven performance standards (types of D & A) and their corresponding verification criteria established by the Maternal and Child Health Integrated Program (MCHIP) [42]. A total of 22 verification criteria for disrespect and abuse were used. For independent variables, questionnaires were adapted after reviewing various literature [16, 17, 43–49]. Generally, the tools consists of three sections; the first section was used to assess socio-demographic characteristics of the mother, the second section was used to assess obstetric and individual-related factors of participants and the third section was used to assess categories of disrespect of women during labor and delivery.

The data were collected by trained collectors using a pretested, structured questionnaire in a face-to-face exit interview from the postnatal care unit. The questionnaire was translated to the local language (Amharic) by language experts before the actual data collection period. Six diploma-holding midwives and three BSC nurses were trained as data collectors and supervisors respectively. The data collectors were assigned to health facilities where they collected the data on each respective participant according to the orientation they had been given on data collection procedures; supervisors and investigators checked the processes and questionnaire responses for consistency and completeness.

### Data processing and analysis

Before starting the actual data collection processes, to assure the data quality, high emphasis was given to designing data collection instruments during and after data collection. Questionnaires were developed in English, translated to Amharic, then translated back into English to ensure consistency. Six female diploma-holding midwives were selected to collect the data, and three BSC nurses were recruited as supervisors from non-study area health facilities. One day of training was given to data collectors and supervisors on the aim of the study, the content of the questionnaire, how to ensure confidentiality and privacy and the techniques of the interview. Then, the questionnaires were pre-tested on 5% (19) of the total sample size in the Billi Health Center from outside of study area. After pre-testing, no further adjustments to the data collection tools were made because there were no findings to do so. During data collection process all of the questionnaires were checked for completeness and accuracy onsite.

The collected data were compiled, checked for any inconsistency and missed value, coded, and entered into Epi-data version 4.6 software and exported into SPSS version 25 for data management and analysis. The data were cleaned for missing values through running frequencies and crosstabs. The findings of this study were described using frequencies, percentages, tables, and graphs. Variables with p-value less than 0.25 in bivariable logistic regression were fitted to multivariable logistic regression model. The Hosmer and Lemeshow test was used to check the final model fitness (p-value was > 0.05). Adjusted odds ratio with 95% confidence interval was computed to assess the degree of association between the outcome and independent variables. Finally, variables with a p-value less than 0.05 in the multivariable logistic regression were considered to have a statistically significant association with the outcome variable.

### Ethical considerations

Ethical clearance was obtained from Wollo University, College of Health Sciences, and School of Public Health Ethical Review Committee (ERC). A etter of permission to conduct the study was obtained from the administrative office of Borena District Health Office. A letter of permission was also obtained from South Wollo Zonal Health Department and given to the district and health facilities. Written informed consent was obtained from all participants before data collection. They were informed that participating in the study was voluntary and their right to withdraw from the study at any moment during the interview was assured. No personal identifiers were used on data collection forms. The recorded data were not accessed by any third person except the principal investigator, and was kept confidentially and anonymously.

## Results

### Socio-demographic characteristics of study participants

A total of 369 postnatal women were interviewed with a response rate of 98.7%. About 104 (28.2%) of participants were between 25–29 years old with a mean (±SD) age of 30.05 (±6.42) years. A majority of the women 254 (68.8%) were married and about 219 (59.3%) were Muslim and more than half (62.1%) were housewives by occupation. About 194 (52%) of the women had never attended any formal education whereas nearly one-fifth (21.7%) had attended college and above (Table 1).

**Table 1:** Socio-demographic characteristics of participating women in Borena District, Amhara Region, Ethiopia, 2020 (n = 369).

Variable	Category	Frequency (n)	Percent (%)
Age	15–19	11	3.0
	20–24	64	17.3
	25–29	104	28.2
	30–34	92	24.9
	≥ 35	98	26.6
Marital status	Married	254	68.8
	Single	48	13.0
	Divorced	34	9.2
	Widowed	25	6.8
	Separated	8	2.2
Religion	Muslim	219	59.3
	Orthodox	145	39.3
	Protestant	5	1.4
Ethnicity	Amhara	354	95.9
	Oromo	15	4.1
Educational status	Unable to read and write	85	23.0
	Able to read and write	109	29.5
	Primary school (grades 1–8)	48	13.0
	Secondary school (grades 9–12)	47	12.7
	College or above	80	21.7
Occupation	Housewife	229	62.1
	Government employee	72	19.5
	Private business	23	6.2
	Student	29	7.9
	Other*	16	4.3
Residence	Urban	224	60.7
	Rural	145	39.3
Wealth index	Poor	147	39.8
	Medium	74	20.1
	Rich	148	40.1

Note:

* = daily laborer, farmer.

### Obstetric history of participants

The majority of the participants 346 (93.8%) had ANC follow up during their most recent pregnancy from those, more than half (51.2%) had ANC visit at the health center and about 210 (56.9%) attended all ANC visits (≥4 visits). 188 (50.9%) of women had delivered at a health center for their most recent previous pregnancy, 174 (47.2%) women were allowed their choice to have families/companions enter the labor room during labor and delivery and the majority (64%) of participants had a history of previous delivery at a health facility. The majority (55%) of participants were delivered by spontaneous vaginal delivery for their most recent delivery, and of them, 29% of women had developed some form of complications during delivery/immediately after delivery (Table 2).

**Table 2:** Obstetric history of women in Borena District, Amhara Region, Ethiopia, 2020 (n = 369).

Variables	Category	Frequency (n)	Percent (%)
ANC visit	YES	346	93.8
	NO	23	6.2
Place of ANC visit	Hospital	142	38.5
	Health center	189	51.2
	Both hospital and HC	15	4.1
Number of ANC visit	≥ 4 visits	210	56.9
	< 4 visits	136	36.9
Parity of the women	One	96	26.0
	Two	102	27.6
	Three	69	18.7
	Four	53	14.4
	Five and above	49	13.3
Who made health decisions in the household	Husband	95	25.7
	Wife	82	22.2
	Both	178	48.2
	Families/parent	14	3.8
Type of health facility	Hospital	181	49.1
	Health center	188	50.9
Presence of companions at birth	Yes	174	47.2
	No	195	52.8
History of previous delivery in a health facility	Yes	236	64.0
	No	133	36.0
Sex of health care providers	Female	129	35.0
	Male	127	34.4
	Both male and female	113	30.6
Type/mode of delivery	SVD	203	55.0
	Vacuum/forceps delivery	72	19.5
	Cesarean section	35	9.5
	With episiotomy	59	16.0
Time of delivery	During day time	170	46.1
	During night time	199	53.9

### The magnitude of disrespect and abuse during facility-based childbirth

**Fig 1:**
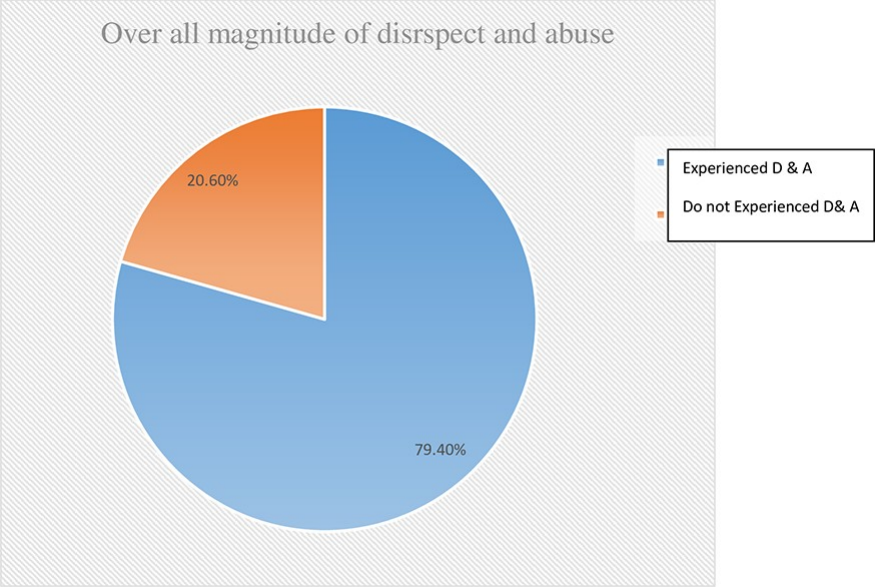
Overall magnitude of disrespect and abuse (D & A) during facility-based childbirth in Borena District, Amhara region, Ethiopia, 2020 (n = 369).

The results of this study showed that the overall magnitude of disrespect and abuse was 79.4% at 95% CI: (77%, 82%) where four out of five women had experienced at least one form of disrespect during facility-based childbirth (Fig 1).

### Categories of disrespect and abuse during facility-based childbirth

**Fig 2:**
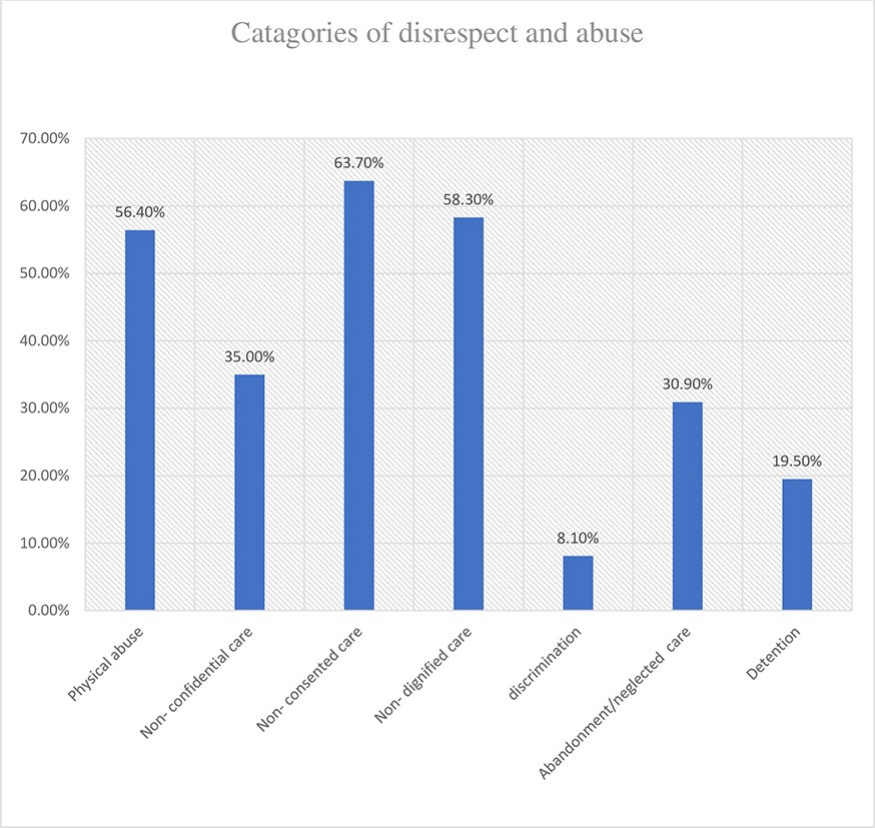
Percentage of each category of disrespect and abuse during facility-based childbirth in Borena District, South Wollo, Amara region, Ethiopia, 2020 (n = 369).

All seven categories of disrespect and abuse with respective standard verification criteria were reported by women in the study. The most reported type of disrespect and abuse was non-consented care 63.7% at 95% CI: (58%, 69%). Under these categories, the most frequently experienced forms of D & A were were having providers who did not introduce themselves or greet the women and her attendant(s) (56.1%), and having providers who did not encourage women or her companion to ask questions (53.1%). The second and third most reported types of disrespect were non-dignified care 58.30% at 95% CI: (53%, 63%), and physical abuse 56.40% at 95%CI: (51%, 61%). Under categories of non-dignified care and physical abuse, the most reported violated criteria were a health care provider’s verbal abuse/insulting language during labor or delivery (40.1%), and not allowing a woman’s companion to enter the delivery room (39.6%). Other types of D & A reported were non-confidential care 35% at 95% CI: (30%, 4%), neglected care 30.9% at 95% CI: (26%, 36%), and detention 19.5% at 95% CI: (15%, 24%). About 8.1% of the women experienced discrimination during facility-based childbirth; for almost half (3.8%) of this group, it was due to their HIV positive status (Table 3) and (Fig 2).

**Table 3:** Magnitude of disrespect and abuse by verification criteria during facility-based childbirth in Borena District, South Wollo, Amhara region, Ethiopia, 2020 (n = 369).

Category and type of disrespect and abuse during labor or delivery	Experience	
Physical abuse	YES %	NO%
Health provider physically hit or slapped, pinched /pushed mother	83 (22.5)	286 (77.5)
Health provider verbally insulted mother	148 (40.1)	221 (59.9)
Mother restricted from drinking any fluid unless medically necessitated	46 (12.5)	323 (87.5)
Birth attendant(s) pushed tummy down to deliver the baby (used fundal pressure)	95 (25.7)	274 (74.3)
Birth attendant(s) allowed mother to move around (ambulate) during the course of the labor	143 (38.8)	226 (61.2)
Non-confidential care		
Health providers used curtains or other physical barriers to maintain privacy	103 (27.9)	266 (72.1)
Health providers discussed mother’s private health information such that others could hear it	57 (15.4)	312 (84.6)
Person(s) other than the care providers were allowed into the birthing room who could observe mother while naked on the bed	74 (20.1)	295 (79.9)
Non-consented care		
rovider did not introduce themselves or greet mother	207 (56.1)	162 (43.9)
providers did not encourage the mother and her companion to ask questions	196 (53.1)	173 (46.9)
provider did not respond to mother’s question with politeness	180 (48.8)	189 (51.2)
provider did explain what was being done and what to expect during care	176 (47.7)	193 (52.3)
Health care provider did not obtain consent or permission before a procedure	178 (48.2)	191 (51.8)
Non-dignified care		
Health care providers shouted at or scolded mother	146 (39.6)	223 (60.4)
Care provider blamed mother for getting pregnant or for shouting due to the pain of labor	101 (27.4)	268 (72.6)
care providers allowed mother’s companion into delivery room	189 (51.2)	180 (48.8)
Discrimination		
Health care providers discriminated by age, ethnicity, religion, and/or being rural	23 (6.2)	346 (93.8)
Health care providers discriminated against mother because of HIV+ status	14 (3.8)	24 (6.5)
Abandonment/neglect of care		
Mother left alone for a period of time without care provider nearby while were in labor	110 (29.8)	259 (70.2)
Mother gave birth alone because the care providers were not present	17 (4.6)	352 (95.4)
Mother encountered a life-threatening condition for which she shouted for help but could not get anyone reached you in time	72 (19.5)	297 (80.5)
Detention in a health facility		
Mother was detained or confined in health facility against her will	72 (19.5)	297 (80.5)

### Factors associated with disrespect and abuse of women during child birth

In the multivariable logistic regression model wealth index, type of health facility, companions/families entering labor & delivery room based on woman’s choice, and facing a labor complication were significantly associated with disrespect and abuse after controlling for the confounding effects of all possible factors at P-value<0.05.

Respondents with the category of poor for wealth index were 3.27 times more likely to be disrespected and abused as compared to those women who were in the richest category [AOR = 3.27; 95%CI: (1.47, 7.25)]. Women who delivered in public health centers were nearly two times more likely to be disrespected and abused than those who gave birth at a public hospital [(AOR = 1.96; 95%CI: (1.01, 3.78)]. Respondents whose companions/attendants were encouraged and allowed to enter the labor and delivery room were 95% less likely to be disrespected and abused than those women whose companions were not allowed [(AOR = 0.05; 95%CI: (0.02, 0.12)]. Moreover, women who were facing a complication in a public health facility were 2.65 times more likely to be disrespected and abused than those women who did not face complications [AOR = 2.65; 95%CI: (1.17, 5.99)] (Table 4).

**Table 4:** Factors associated with disrespect and abuse among women who delivered in public health facilities of Borena District, Amhara region, Ethiopia, 2020 (n = 369).

Variables	Disrespect and abuse		COR at 95% CI	AOR at 95% CI
	YES (%) NO (%)			
**Marital status**				
Single	45(15.4)	3(3.9)	1.75(0.43, 7.14)	2.18(0.42, 11.27)
Married	188(64.2)	66(86.8)	0.33(0.15, 0.76)**	0.64(0.24, 1.74)
Other^1^	60(20.5)	7(9.2)	1	1
**Educational status**				
No formal education	71(24.2)	14(18.4)	1	1
Primary education	145(49.5)	30(39.5)	0.95(0.48, 1.91)	1.97(0.83, 4.72)
Secondary or above	77(26.3)	32(42.1)	0.47(0.23, 0.96)*	1.70 (0.29, 1.67)
**Residence**				
Rural	124(42.3)	21(27.6)	1.92(1.11, 3.34)*	0.70(0.29, 1.73)
Urban	169(57.7)	55(72.4)	1	1
**Wealth index**				
Poor	132(47.1)	15(19.7)	3.72(1.96, 7.06)**	3.27(1.47, 7.25)**
Medium	57(19.5)	17(22.4)	1.42(0.74, 2.71)	1.25(0.56, 2.77)
Rich	104(35.5)	44(57.9)	1	1
**ANC visit**				
YES	271(92.5)	75(98.7)	0.16(0.02, 1.24)	0.27(0.03, 2.49)
NO	22(7.5)	1(1.3)	1	1
**Type of health facility**				
Health center	158(53.9)	30(39.5)	1.79(1.07, 3.00)*	1.96(1.01, 3.78)*
Hospital	135(46.1)	46(60.5)	1	1
**Presence of companions**				
YES	105(35.8)	69(90.8)	0.06(0,03, 0.13)**	0.05(0.02, 0.12)**
NO	188(64.2)	7(9.2)	1	1
**Mode of delivery**				
CS / Assisted delivery	138(47.1)	28(36.8)	1.53(0.91, 2.57)	0.99(0.49, 2.04)
SVD	155(52.9)	48(63.2)	1	1
**Faced complication**				
YES	97(33.1)	10(13.2)	3.27(1.61, 6.63)**	2.65(1.17, 5.99)*
NO	196(66.9)	66(86.8)	1	1
**Time of delivery**				
During day time	142(48.5)	28(36.8)	1.61(0.96, 2.71)	1.46(0.78, 2.74)
During night time	151(51.5)	48(63.2)	1	1

Note:

* indicate that (p value = <0.05, and

** indicated (p value = <0.01).

## Discussion

Among women who delivered at a health facility, nearly four out of five (79.4%) experienced at least one type of disrespect and abuse. Wealth index, type of health facility, companions/families allowed to enter labor room based on a woman’s choice, and facing a complication were found to be significantly associated with the outcome variable. It indicated that there is a high magnitude of disrespect and abuse of women during maternity care in this study area, a situation that needs urgent interventions. This result is consistent with a study conducted in Addis Ababa, Ethiopia that showed that the prevalence of disrespect and abuse was 78% [19]. The reason for the similar findings may be that the standard verification criteria in both studies are very similar and both studies focus on the same kinds of public health facilities.

On the other hand, this study’s finding is lower than that of studies conducted in southern Ethiopia 91.7% [50], Tanzania 85.3% [51], Malawi 93.7%[52], and Pakistan 97% [53]. There may be three possible reasons for that difference. First, there were larger sample sizes and time variation in those previous studies. Second, there was a study population difference in that the previous studies excluded women who had delivered by cesarean section and evidence has shown that women who deliver by cesarean section are less likely to experience disrespect and abuse than those who delivered vaginally [47, 54]. This could be because health care providers tend to devote more attention to women indicated for cesarean delivery, in particular, being more likely to strictly attend to informed consent of this group compared to that of women who delivered vaginally. Third, there were variations in data collection, in that the previous studies used a direct observational method, while the current study used an exit interview method. Interviewing participants may be affected by recall bias while data collected via direct observation may be more reliable for recording every activity.

The magnitude of D & A found by this study was higher than found in Addis Ababa, Ethiopia 17.6% [43], northern Ethiopia 75.1% [16], Kenya 20% [55], northwestern Tanzania 73.1% [56], and urban Tanzania 15% [57]. The possible reasons may be variation in sample size [47, 55, 56, 58]; variation among standard verification criteria to measure disrespect and abuse [43, 55, 56, 59, 60]; study setting difference; health care quality implementation program differences; socio-cultural and socio-economic status disparity; geographical variation, and time variation.

This study showed that poor respondents were three times more likely to be disrespected and abused than those women who had a high wealth index level (rich). This result was consistent with a previous studies conducted in Ethiopia [17, 43, 61, 62] and in Gujrat Pakistan [5] that showed that poor women were more likely to experience disrespect and abuse during facility-based maternity care at facility based childbirth. The reason may be that rich women might be more respected and cared for than poor women by facility-based health care providers.

Those women who delivered in a public health center were nearly twice as likely to be disrespected and abused than those women who delivered in a public hospital. Reasons for this finding by the current study may be that heath service deliver in low-level settings is affected by factors such as a lack of standards; a lack of leadership/supervision; poor clinical care; and a lack of multidisciplinary teams and training compared to the higher-level setting [2]. In contrast to findings of this study, some previous studies done in Ethiopia [17, 18] and northern India [49] showed that women who delivered at a public hospital reported at least one more type of disrespect and abuse than those who gave birth at a health center. The possible reason for this may be that in hospitals with higher case numbers and overflowing referrals of complicated cases leading to overcrowding, care providers may be pushed to perform abusive care in contrast to in health centers where there may be relatively fewer complicated cases and care providers are less stretched.

Respondents whose companions/supporters were encouraged and allowed to enter the labor and delivery room based on her choice were 95% less likely to be disrespected and abused than those women whose companions/supporters were not allowed to enter the labor room. This finding is supported by similar studies done in Ethiopia [63–65], Kenya [56], and Malawi [52]. The reasons may be that families of laboring women have an important role in reducing the impact of D & A. The presence of a companion during labor and delivery is a very effective approach to minimizing D & A by health professionals; it also makes women happier, psychologically ready for good motherhood. On the other hand, women felt disappointed with the health system when they were not allowed to have a support person in the delivery room, leading to their overall dissatisfaction with and reluctance to receive facility-based maternity care.

Women who were facing a complication during delivery in a public health facility were twice as likely to be disrespected and abused as those who did not face any complications. This finding is consistent with a similar study done in northern Ethiopia [48], Tanzania [56], and India [49].

The possible reason may be that women who develop complications have to stay a longer time in the health facility, increasing her contact time with care providers and the opportunities for D & A. These women might suffer secondary pain related to the complication in addition to labor pain. Facing complications can lead to stressed labor and poor maternal health effects as a result of frequent and unnecessary examinations by multiple care providers. Neglect of women by health care providers during labor and delivery or poorly skilled attendants may be a consequence of a poor health system.

This study showed that 30.9% of women were neglected during labor and delivery care; about 29.8% left alone without care provider while in labor and needing help and 4.6% gave birth by themselves because the care providers were not around. It showed the low commitment of health care providers. Overall, these findings show a violation of the right to health, the barrier to institutional health care delivery, and factors related to mothers receiving poor quality health care.

### Limitations

The limitations of this study were its quantitative design, being based on interview alone, excluding observational form of data collection, and its cross-sectional design that made it difficult to establish temporal relationships between explanatory variables and the outcome variable.

## Conclusion

The magnitude of disrespect and abuse was very high (79.4%) as four out of five of women experienced at least one type of disrespect and abuse during facility-based childbirth. Wealth index, type of health facility, women’s companions/families allowed to enter the delivery room, and facing complications during labor and delivery were found to be significantly associated with the experience of disrespect and abuse. Health care managers and health professionals need to develop interventions to reduce the impact of these factors on women’s health, and to improve compassionate and respectful care by health care providers as part of a strategy for the delivery of quality health care.

## Acronyms/abbreviations

ANC: antenatal care
AOR: adjusted odds ratio
CI: confidence interval
COR: crude odds ratio
CRC: compassionate and respectful caring
CS: cesarean section
D & A: disrespect and abuse
EDHS: Ethiopian Demography and Health Survey
ERC: Ethical Review Committee
FMOH: Federal Minister of Health
HIV: human immunodeficiency viruses
MCHIP: Maternal and Child Health Integrated Program
MMR: maternal mortality rate
RMC: respectful maternal Care
SD: standard deviation
SE: standard error
SPSS: Statistical Package for Social Sciences
SSA: Sub-Saharan Africa
SVD: spontaneous vaginal delivery
WHO: World Health Organization
