# Expression of Concern: Magnitude and associated factors of disrespect and abusive care among laboring mothers at public health facilities in Borena District, South Wollo, Ethiopia

**DOI:** 10.1371/journal.pone.0329311

**Published:** 2025-07-30

**Authors:** The PLOS One Editors

The *PLOS One* Editors issue this Expression of Concern to alert readers that this article [1] was identified as one of a series of submissions for which we have concerns about authorship and adherence to the journal’s first and second publication criteria. We regret that these issues were not addressed prior to the article’s publication.
